# The Structural Characteristics of Economic Network and Efficiency of Health Care in China

**DOI:** 10.3389/fpubh.2021.724736

**Published:** 2021-08-23

**Authors:** Kuang-Cheng Chai, Yang Yang, De-Cong Xie, Yang-Lu Ou, Ke-Chiun Chang, Xiao Han

**Affiliations:** ^1^Business School, Guilin University of Electronic Technology, Guilin, China; ^2^School of Economics and Management, Wuhan University, Wuhan, China; ^3^Institute of Central China Development, Wuhan University, Wuhan, China

**Keywords:** efficiency of health care, network structure, social network analysis, cluster analysis, medical resources

## Abstract

With the rapid development of the economy of China, the interactivity between provinces and the mobility of the population is increasing. Some patients who could have received the same treatment in their residential areas still choose to receive services in areas with higher economic development and concentrated high-quality medical resources, resulting in a huge waste of medical resources. Blindly increasing medical resources everywhere does not necessarily increase the output effectively. In this study, the data envelopment analysis (DEA) model, social network analysis (SNA), cluster analysis, and regression analysis are used to analyze the structural characteristics of the economic network structure and efficiency of health care in China. The results show that indegree and eigenvector centrality have a significant positive correlation with the efficiency of health care, and the clustering coefficient has a significant negative correlation with the efficiency of health care in China. This study uses a k-means algorithm to classify 31 provinces into three groups and extract their characteristics. As for the supply of health care resources, the government should command and dispatch the resources in the whole country through a top-down design based on the characteristics of each province.

## Introduction

With the development of the economy of China, in recent years, the construction of public infrastructure has improved a lot. Also, the right of people to health needs should be guaranteed with high quality, which calls for higher requirements for the allocation of medical resources and services. At present, China has elevated health to an important position in its strategic development toward the policy of “Healthy China,” which paid a lot of attention to public fitness, food safety, environmental pollution, and public health emergencies. The medical and health services of China have developed rapidly (1). Although government investment continues to increase in medical service, and health service supply capacity and the level of the quantity received a significant boost, the medical and health care of China still faces problems, such as imbalanced levels of regional medical development and low efficiency of health care.

The increase in medical resources corresponds to an increase in social security, tranquility, safety, and welfare of a country. It helps severely ill people to shorten the recovery time and return to work (2), thus improving the overall labor efficiency and achieving the purpose of promoting sustainable economic development. However, excessive medical service input and rapid increase in medical service fee standards have led to the national cost crisis. Problems such as the rapid increase in health care costs (as Japan faced in the past) and severe government fiscal deficits also exist. Therefore, blind investment in limited medical resources is uneconomical. Many scholars also believe that medical and health expenditures can be greatly reduced, although better schemes to reduce costs have not been deduced yet (3). Owing to the large span of the regions of China, poor economic interaction between regions, lack of resource information sharing, backward diffusion of scientific and technological achievements, and other obstacles, the economic development gap between the regions of China is widening with each passing day. The unbalanced development of the regional economy of China actuates the “inverse care law” of medical and health services, which is mainly manifested as the relatively small distribution of medical resources in poor areas and poor geographical accessibility of medical services owing to the large urban-rural and regional differences. There is an inverse correlation between quality medical and health services and the health needs of people, indicating that people in poor areas have higher health needs (4). Therefore, it is of vital importance to rationally allocate national medical resources according to the economic connections between provinces, and to improve the quality and efficiency of health care in underdeveloped areas to meet the health needs to the maximum extent.

This study has the following contributions: First, the economic network of China is constructed from the perspective of social network analysis (SNA), and the efficiency of health care is evaluated by the data envelopment analysis (DEA) method. Second, this study tests the effect of the characteristics of economic network structure, such as degree centrality, the clustering coefficient, and eigenvector, on the efficiency of health care in China. The regression result can provide new empirical evidence for relevant fields. Finally, 31 provinces in China are grouped into three categories by using the k-means algorithm. Thus, according to the characteristics of each category, combined with the results of regression analysis, more targeted suggestions for management are put forward.

## Literature Review

Health care is very important to population health. As an important part of health care, medical resources play a key role in keeping people healthy. Investment in health resources will reduce the increasing mortality rate, control infectious diseases, and improve the health of mothers and children (5). There is a negative correlation between health resources and mortality (6, 7); medical advances can extend life spans (8) and more directly and quickly meet individual medical needs (9). In the 1990s, Sherman (10) first applied DEA in the field of health care to evaluate whether resources are effectively utilized by relative efficiency. Scholars have extensively applied the DEA model to the medical and health system and researched the efficiency of hospitals in a variety of ways. Nayar et al. (11) evaluated the efficiency differences of different types of hospitals. Hamidi (12) collected the balanced panel data of government hospitals over 6 years and used the stochastic frontier approach (SFA) model to measure the technical efficiency of government hospitals through the input variables of beds, doctors, nurses, and non-medical personnel in the hospital and the output variables of the total number of inpatients and outpatients. Li et al. (13) collected the relevant data of 12 county-level public hospitals in Anhui Province, China, from 2010 to 2015. The results showed that the technical efficiency of county-level hospitals in China was low, and the surplus medical resources in poor areas were much higher than those in non-poor areas. The overall operating efficiency of hospitals in poor areas was very low, and regional economic differences affected the performance of hospitals. Jiang et al. (14) used the DEA model to calculate the efficiency of 1,105 sample hospitals in 31 provinces of China and confirmed the health care efficiency in China was relatively low, with the highest efficiency in Eastern China and the lowest in Western China. Owing to the emphasis on the construction of large urban hospitals and the neglect of basic medical service institutions, and on account of the blind pursuit of interests by hospitals and excessive expansion of hospital scale, the current medical resource use efficiency of China is not high and there is a lot of wastage. To sum up, the DEA model is widely used in medical and health systems in related studies.

Many previous studies have focused on the relationship between economy and medical resource allocation. Economic crisis can lead to an increase in the unemployment rate and a decrease in medical resources (15). Moreover, a poor economy reduces the input of government fiscal revenue to public health and the poor population has less access to health resources. From an international viewpoint, with the improvement of national economic development, the expenditure on medical and health will also increase (16), and there is a long-term equilibrium relationship between the two. The developed regions of China have strengthened the less-developed regions through long-term cooperation and transformation, which include support for health technicians, equipment maintenance, and funding. Local governments have reinforced regional health planning, including the integration, and sharing of medical resources among regions while focusing on weak links such as investment in rural and community health resources. Based on the actual conditions, all localities should improve the medical system while achieving all-round development and gradually implement and improve the allocation of health resources nationwide. The measures to further meet the health service needs of local people include increasing the total number of health personnel, evaluating their service quality, and optimizing the allocation of health personnel resources in areas with low economic development (17). The problem of insufficient reserves of conventional medical resources becomes exposed after the outbreak of public health emergencies and can be resolved only through the “reconfiguration” of medical resources. SNA involves studying social relationships and structures, focusing on the internal networks between different social actors (18). Developed in the 1930s, SNA is used to analyze the relationships between people. It has developed rapidly and combined with mathematics, statistics, computer science, and other disciplines to become an interdisciplinary analytical method. It is mainly composed of points and lines. It describes the interactive structural relations between individuals through quantitative indicators and reflects the characteristics of the entire network structure and the position of individual objects in the network structure. The structure in this study could be behavioral structure, political structure, social structure, or economic structure. The regional economy is open; any region can have an economic connection and economic penetration with other regions through the flow of resource elements, such as population, materials, capital, technology, and flow of information through economic agglomeration and spillover. Therefore, it is of great significance to use SNA to quantify the economic conditions and test the effect of structural characteristics of economic networks on the efficiency of health care in China. In the age of big data, the statistical method in exploratory data analysis such as cluster analysis is often used to discriminate objects by similarity or difference. Since the introduction of k-means, due to its effectiveness, simplicity, and speed in data analysis, the k-means algorithm has been widely used in various fields, resulting in different management practices. Based on the result of DEA and SNA analysis, this study uses cluster analysis to expand the depth of the research and puts forward targeted suggestions accordingly.

## Methods and Measurements

### Data Collection

All data are derived from authoritative sources such as the China Health Statistical Yearbook, the relevant regional statistical yearbook, and the statistical bulletin (2014–2018). The fixed assets and human resources of health care should all be considered when considering the current situation of efficiency of health care in a certain area. According to the requirements of the DEA model, the 31 provinces in China are taken as decision units to evaluate health care in each province each year. The number of medical and health institutions, the number of hospital personnel, and the number of beds in medical and health institutions are selected as the input indexes, and the hospital bed utilization rate and total outpatient visits are selected as output indicators based on the input and output indexes set in the existing literature.

### Variable Measurement

#### DEA-Malmquist

This study uses the DEA method to measure the efficiency of health care in various provinces of China. DEA is a non-parametric technical efficiency analysis method based on a relative comparison between the evaluated objects. DEA has a wide application range, a relatively simple principle, and does not need to consider the dimensionality of data. Therefore, it has a special advantage in analyzing the input-output efficiency of the macroeconomic system. According to the requirements of the DEA model, the 31 provinces in China are taken as decision units to evaluate the efficiency of health care in each province each year. Based on the input and output indexes set in the existing literature, the number of medical and health institutions, the number of hospital personnel (health technicians, other technical personnel, management personnel, workers, and technicians), and the number of beds in medical and health institutions are selected as the input indexes. The hospital bed utilization rate and total outpatient visits are selected as output indicators. The smaller the efficiency value, the greater the degree of resource waste under the existing resource input. Caves et al. (19) applied the Malmquist index to the production front efficiency analysis method to measure efficiency and constructed an efficiency evaluation index by using a distance function. To better understand the dynamic distribution of medical resources in each province of China, the Malmquist index is used to calculate and analyze the medical resources in each province, which can solve the problem of relative efficiency change. Total factor productivity (TFP) change refers to the overall efficiency of production activities in a certain period of time. TFP change can be further decomposed into technical change and efficiency change. Technical change stands for growth through technological progress, while efficiency change represents the extent to which existing technology is effectively utilized, assuming that the overall return to scale remains unchanged. The efficiency change can further be decomposed into pure efficiency change that measures the changes in the closeness of the firm to the frontier and scale efficiency change, which indicates if the movement inside the frontier is in the right direction to attain the scale efficiency.

#### Gravity Model and Economic Network

The gravity model is derived from the Newtonian law of gravity in physics and it is widely used to describe the economic relationships between cities (20). The gravity model can not only reflect the radiating capacity of the economic center province to the surrounding area but also reflect the degree of acceptance of the surrounding area to the radiating capacity of the economic center. In this study, the standard form of the gravity model was adopted, that is, the strength of the economic connection between two provinces is proportional to the mass of the region and inversely proportional to the square of the distance between them. The product of gross domestic product (GDP) and population of the province is used to describe the quality of the province, and the distance between the provincial capitals is used to describe the distance between the two provinces.

The node assignment matrix of the regional economic network map should be established to obtain the index of the urban economic network map and its network density. The average value of each line of the calculated gravity value matrix is taken as the threshold value of the line. If it is greater than the threshold value, the economic connection between provinces is set as 1; if it is less than the threshold value, the economic connection is set as 0.

$$\begin{array}{l} R_{ij}=R_{ji}=\frac{\sqrt{GDP_{i}P_{i}}\sqrt{GDP_{j}P_{j}}}{d_{ij}^{2}} \end{array}$$

**Figure 1:**
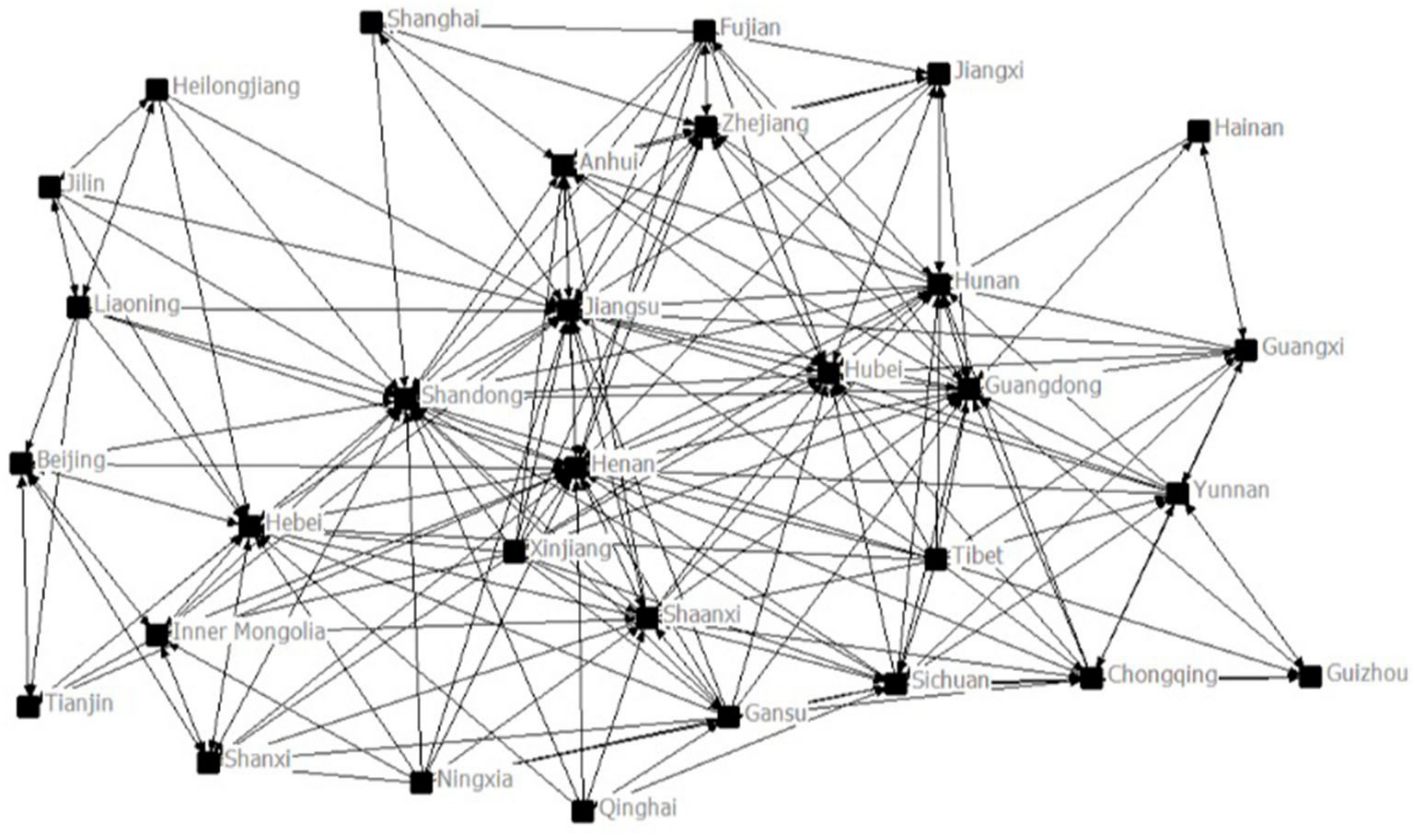
The economic network construction of China.

Among them, R_*ij*_ represents the economic attraction between two regions (10,000 yuan ^*^ 10,000 people/km^2^); GDP_*i*_ and GDP_*j*_ represent the GDP of region *i* and region *j*, respectively; P_i_ and P_j_ represent the population of region *i* and region *j*, respectively; and d_*ij*_ is the geographical distance between regions *i* and *j*. Both GDP and population data are derived from the China Statistical Yearbook for the corresponding years and the geographical distance between regions is expressed as a straight-line distance between their provincial capitals. Using UCINET, we create a directed graph (Figure 1), the economic network composed of provincial central cities, and the structural characteristics of the economic network are analyzed by calculating the network density, degree centrality, and eigenvector centrality.

Degree centrality: In the economic network of China, the more direct connections between a province and other provinces exist, the more central the province is in the network, the greater “power” it has in the network. This index reflects the economic communication ability between node provinces and other provinces and the degree of centrality in the economic network and reflects the core competitiveness of the provinces. Centrality can be divided into indegree and outdegree centralities according to the direction of regional economic relations. Indegree centrality reflects the extent to which a province is influenced by other provinces and measures the economic agglomeration ability of a province. The formula for indegree centrality is as follows:

$$\begin{array}{l} Indegree_{i}=\overset{n}{\underset{j=1}{\sum }}F_{ij} \end{array}$$

N is the number of nodes, and F_*ij*_ is the economic connection strength between provinces *i* and *j*.

The clustering coefficient: It refers to the ratio of the actual number of edges to the maximum possible number of edges for a small network composed of all nodes in the neighborhood of this node. The clustering coefficient of a node is between 0 and 1. The higher the value, the closer the connection between other nodes connected to the node. The formula is as follows:

$$\begin{array}{l} Clustering=\frac{2E_{i}}{k_{i}\left(k_{i}-1\right)} \end{array}$$

In the formula, K_*i*_ is the number of edges connecting node i with other nodes, namely, the degree of node *i*, and E_*i*_ is the number of edges that exist between nodes of K_*i*_.

Eigenvector centrality: It measures the importance of other provinces associated with the province. The higher the centrality of the feature vector of a province, the higher the node status of other provinces directly related to it in the provincial network.

$$\begin{array}{l} Eigenvector_{jt}=\lambda \overset{N_{t}}{\underset{i=1}{\sum }}A_{ijt}Eigenvector_{jt} \end{array}$$

where N_*t*_ is the total number of nodes in the network and λ is the maximum eigenvalue of the adjacency matrix A_*ijt*_.

## Results

### Individual Network Structure Characteristics

Table 1 displays the individual network structure characteristics of China in 2018. The indegree measures the ability of a province to attract external resources. According to indegree, the key provinces of China have a strong resource attraction in the economic network of the country, with Shandong, Henan, and Jiangsu ranking the top three. Shandong has the highest eigenvector centrality, indicating that provinces directly related to Shandong have a higher node status in the economic network system of China, and the subnetwork quality is composed of provinces directly related to Shandong is the highest. Jiangsu, Henan, Anhui, and other provinces also have high eigenvector centrality, which reflects that these provinces have good relationship circles in the network. The clustering coefficients of Shanghai, Fujian, and Jiangxi are higher, which indicates that the neighboring provinces have a higher degree of interconnection with these provinces.

**Table 1:** Characteristics of the economic network of China in 2018.

**Province**	**Indegree**	**Eigenvector**	**Clustering**
Shanghai	3.00	0.25	0.85
Anhui	12.00	0.82	0.58
Shandong	24.00	1.00	0.28
Jiangsu	22.00	0.93	0.29
Zhejiang	11.00	0.79	0.61
Yunnan	5.00	0.07	0.54
Sichuan	11.00	0.15	0.38
Guangdong	14.00	0.38	0.38
Guangxi	4.00	0.08	0.51
Henan	23.00	0.96	0.30
Hubei	16.00	0.75	0.44
Hunan	13.00	0.47	0.43
Guizhou	4.00	0.06	0.70
Chongqing	7.00	0.10	0.51
Inner Mongolia	5.00	0.16	0.54
Beijing	6.00	0.30	0.67
Tianjin	5.00	0.25	0.70
Shanxi	9.00	0.41	0.56
Hebei	15.00	0.45	0.33
Jilin	9.00	0.21	0.40
Liaoning	2.00	0.01	0.65
Heilongjiang	2.00	0.01	0.55
Shaanxi	2.00	0.01	0.65
Ningxia	1.00	0.01	0.60
Gansu	4.00	0.04	0.45
Jiangxi	6.00	0.44	0.79
Fujian	2.00	0.07	0.83
Hainan	4.00	0.26	0.70
Xinjiang	0.00	0.00	0.51
Qinghai	1.00	0.01	0.67
Tibet	0.00	0.00	0.50

### Malmquist Index Measurement

From Table 2, we can see the Malmquist index and decomposition of 31 Chinese provinces from 2014 to 2018. During the period, the TFP of all 31 Chinese provinces declined. Through further analysis, it is found that the TFP of Shanghai, Guizhou, Beijing, Tianjin, Ningxia, and Tibet are reduced simply because of technological decline. Technological progress is the key factor to promote the improvement of TFP in China. Shanghai, Beijing, and other provinces have relatively high efficiency of health care, while provinces such as Heilongjiang and Tibet have relatively low efficiency.

**Table 2:** Malmquist index summary of province means.

**Province**	**Efficiency**	**Technical**	**Pure efficiency**	**Scale efficiency**	**TFP**
	**change**	**change**	**change**	**change**	**change**
Shanghai	1	0.993	1	1	0.993
Anhui	0.976	0.985	0.977	0.999	0.961
Shandong	0.980	0.988	0.986	0.993	0.967
Jiangsu	0.990	0.993	1.014	0.977	0.984
Zhejiang	0.994	0.988	1	0.994	0.982
Yunnan	0.986	0.986	0.985	1	0.971
Sichuan	0.978	0.987	0.854	1.146	0.965
Guangdong	0.972	0.988	1	0.972	0.960
Guangxi	0.954	0.985	0.876	1.089	0.940
Henan	0.967	0.987	0.971	0.996	0.955
Hubei	0.968	0.987	0.858	1.128	0.955
Hunan	0.946	0.985	0.946	1	0.932
Guizhou	1.010	0.981	1.010	1	0.991
Chongqing	0.960	0.978	0.954	1.006	0.939
Inner Mongolia	0.975	0.978	0.976	1	0.954
Beijing	1.004	0.988	1.004	1	0.992
Tianjin	1	0.978	1	1	0.978
Shanxi	0.987	0.979	0.988	0.998	0.966
Hebei	0.965	0.985	0.969	0.996	0.951
Jilin	0.987	0.976	0.987	1	0.964
Liaoning	0.979	0.986	0.981	0.998	0.965
Heilongjiang	0.954	0.973	0.958	0.995	0.928
Shaanxi	0.975	0.983	0.976	0.999	0.958
Ningxia	1	0.955	1	1	0.955
Gansu	0.962	0.981	0.968	0.994	0.944
Jiangxi	0.954	0.984	0.953	1.001	0.939
Fujian	0.995	0.985	0.996	0.999	0.980
Hainan	0.990	0.956	1	0.994	0.951
Xinjiang	0.998	0.971	1.015	0.983	0.970
Qinghai	0.991	0.949	0.982	1.009	0.940
Tibet	1	0.928	1	1	0.928

### Descriptive Statistics

As shown in Table 3, there is a large gap between the maximum and minimum values of the efficiency of health care, indicating a high regional difference in the efficiency of health care in China. In addition, the difference between the maximum and minimum values of indegree is 25, indicating that the regional development of China is relatively unbalanced, and there is a large gap in economic status between regions.

**Table 3:** Descriptive statistics.

**Variables**	**N**	**Mean**	**S.D**.	**Min**	**Max**
TFP change	155	1.074	0.542	0.280	2.965
Indegree	155	7.690	6.668	0.000	25.000
Eigenvector	155	0.292	0.309	0.000	1.000
Clustering	155	0.541	0.151	0.268	0.850

### Regression Analysis

As shown in Table 4, indegree and eigenvector centrality have a significant positive correlation with the efficiency of health care, while the clustering coefficient has a significant negative correlation with the efficiency of health care in China. The more centrally located the province is in the economic network of China, the greater the efficiency of health care. However, the closer the degree of connection with the critical provinces of the province, the lower the efficiency of medical service data allocation in the region.

**Table 4:** Regression result.

**Variables**	**TFP change**	**TFP change**	**TFP change**
Indegree	0.010^***^	–	–
	(2.850)	–	–
Eigenvector	–	0.210^**^	–
	–	(2.570)	–
Clustering	–	–	−0.266^*^
	–	–	(−1.86)
Constant	0.885^***^	0.895^***^	1.126^***^
	(13.850)	(13.990)	(11.550)
Observations	155	155	155
Region	Control	Control	Control
Year	Control	Control	Control
Adj R-squared	0.765	0.763	0.758

*** *p < 0.01,*

** *p < 0.05,*

* *p < 0.1*.

### Cluster Analysis

**Figure 2:**
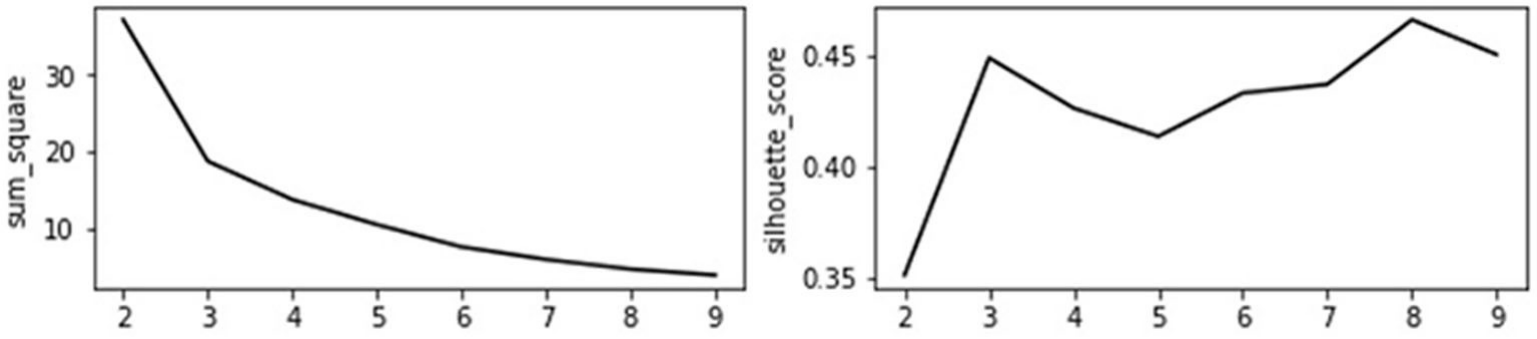
Cluster category evaluation index.

The principal component analysis (PCA) algorithm is first used to extract the features of the structural characteristics of economic network and efficiency of health care. The four variables are divided into two categories and the contribution rate of the characteristic variance is 0.859. The scores of PCA are then used for cluster analysis to group similar instances into clusters. Before determining the grouping category, the evaluation indicators of each grouping are compared. Sum_square represents the sum of the distances from the sample to the nearest cluster center. The smaller the value, the better. The smaller the value, the more concentrated the distribution of the samples among the classes. The higher the score in silhouette, the closer the distance between samples in the same category and the different categories. As shown in Figure 2, it is more appropriate to divide it into three categories.

**Figure 3:**
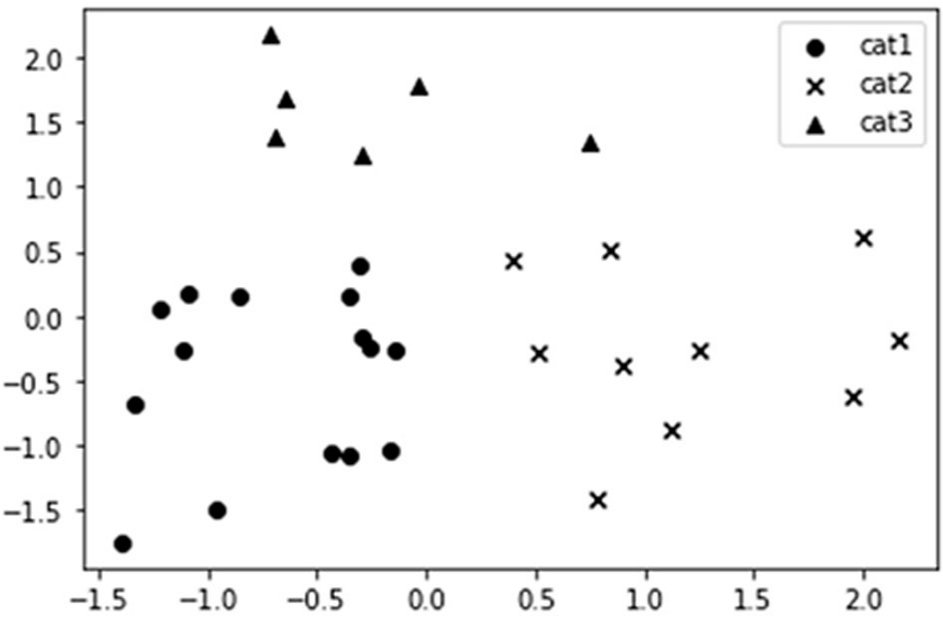
Result of cluster analysis.

In Figure 3, the x-axis is the score of the first component of PCA, and the y-axis is the score of the second component of PCA. The x-axis can be simply understood as the component of the importance of economic network status, and the y-axis is understood as the combination of the efficiency of health care and the network constraint coefficient.

#### Category I: Inner Mongolia, Liaoning, Jilin, Heilongjiang, Jiangxi, Guangxi, Hainan, Chongqing, Yunnan, Xizang, Shaanxi, Gansu, Qinghai, Ningxia, and Xinjiang

Most of these provinces belong to the western and northeast regions, with a sparse population, relatively backward economic transportation, low status of economic networks, and low efficiency of health care. For such areas, the development of medical institution construction projects should be accelerated and the pilot construction of regional medical centers should be promoted. In the face of the lack of bed resources, a plan to increase the number of beds in medical and health institutions should be put forward, and the Chinese government should provide support for policy approval. Steps should be taken to increase financial input and policy preference for medical and health care in resource-poor areas, improve the treatment level of talent in primary medical institutions, further promote the reform of professional titles in primary medical institutions, attach importance to the localization of medical and health talents, consolidate the talent base of primary hospitals, and promote the rational allocation of high-quality medical service resources.

#### Category II: Hebei, Shanxi, Jiangsu, Anhui, Shandong, Henan, Hubei, Hunan, Guangdong, and Sichuan

Most of these provinces belong to the central region, which is an important transportation hub with a superior geographical location. They are also influenced by the radiation and driving effect of the three major economic circles of China and have a high status of the economic network and high efficiency of health care. Such areas should strengthen the pace of medical institution standardization construction and give full play to their medical level advantage to truly build regional medical centers, under the premise of complying with the total bed plan, the size of beds should be increased reasonably according to the change in regional population and medical needs. Measures should be taken to improve the recruitment of public health professionals at the community level, step up efforts to introduce and train high-level public health professionals, establish a multichannel funding guarantee and salary incentive mechanism, allow medical staff in qualified regional medical centers to work in more than one location, and encourage high-quality medical personnel to be recruited.

#### Category III: Beijing, Tianjin, Shanghai, Zhejiang, Fujian, and Guizhou

Due to the preferential policies and their advantages, provinces such as Beijing, Tianjin, and Shanghai have concentrated on advanced diagnosis and treatment technology, equipment, and excellent personnel, with comprehensive development ability, high status in the economic network, and high efficiency of health care. Medical institutions in such areas should study and formulate project construction plans based on the actual situation of the region, integrate existing resources to compensate for shortcomings, strengths, and weaknesses, improve quality, further amplify the radiating and leading role of regional medical centers, balance the distribution of medical resources among regions, and coordinate fairness and efficiency. Under the background of rich bed resources, the size of beds should be strictly controlled while the reasonable increase of beds and strict management should be carried out according to the requirements of regional health planning. Local governments should cultivate domestic high-end medical talents, recruit overseas high-level medical and health talents globally, and lay a talent foundation for the transformation and upgrading of medical centers. The central government should also strive to provide financial support for the national equalization of medical and health services, increase the transfer payment to underdeveloped areas, overcome the non-equalization of medical services caused by the differences in financial capacity among provincial areas, and finally realize the synchronous improvement in the quality and efficiency of health care.

## Conclusions and Suggestions

In this study, the gravity model and SNA are combined to study the structural characteristics of the economic network of China, the DEA-Malmquist method is used to evaluate the medical efficiency, and regression analysis is used to study the relationship between them. The results showed that the indegree and eigenvector have a significant positive correlation with the efficiency of health care in China, while the clustering coefficient has a negative correlation with the efficiency of health care. It suggests that the stronger the economic agglomeration ability, the higher the efficiency of health care.

Moreover, to visualize the regional characteristics and put forward targeted suggestions, the study extract features of structural characteristics and efficiency of health care in China by PCA algorithm and classify the provinces by k-means clustering algorithm. According to the result of cluster analysis, the provinces of the first category are mainly distributed in the western and northeast regions where the economy and the efficiency of health care are relatively low. The government should increase the preferential policies in undeveloped areas and raise the salary level of medical institutions in remote areas to attract high-level medical personnel. Provinces of the second category are mostly located in central China and have convenient transportation, these provinces should take advantage of their location and ability to attract capital to rapidly upgrade their economies and serve as hubs to balance regional development. Finally, the third category has a good economy and relatively high efficiency of health care, which should pay attention to foster medical staff of high quality and actively assist economically backward areas.

Through the data analysis and mining in this paper, some suggestions can be put forward. First, the government should increase the construction of transportation especially in remote areas, which can bring investment, talent, technology, and other benefits to regions, and promote industrial interaction among provinces to balance the economic structure of China. In the short term, the medical level in remote areas cannot be rapidly improved, so interprovincial medical treatment should be included in the management of local medical insurance departments. The medical insurance responsibilities, cost-sharing, and dispute settlement mechanisms of the two provinces should be clarified to protect the rights and interests of patients seeking medical treatment in different provinces. Moreover, transportation hub areas such as the central regions in China should give full play to their regional advantages, rationally plan the allocation of medical resources, and strive to serve more people with better service. During the epidemic period, a large medical resource reserve center should be established here to facilitate assistance to surrounding areas and save transportation costs. Finally, the fairness and accessibility of the health service system should be properly determined by the government with full consideration of various factors such as the level of regional social and economic development, population status, geographical conditions, distribution of health resources and services, the demand of residents, and utilization of health services.

As the number of cases of COVID-19 is increasing worldwide, any country may face the prospect that the number of critically ill patients will outpace the available care resources. Correspondingly, the government should avoid the shortage of medical resources and formulate relevant policies to rectify the areas with low economic network status and low efficiency of health care. The state can strengthen macro-control to promote mutual coordination of materials and human resources in various regions, enhance and balance regional economic development, and improve the efficiency of health care at the same time.

## Data Availability Statement

The raw data supporting the conclusions of this article will be made available by the authors, without undue reservation.

## Author Contributions

Ku-CC: project administration. YY, D-CX, and Y-LO: investigation and validation. Ku-CC, YY, D-CX, and Y-LO: formal analysis. Ku-CC, YY, and Ke-CC: methodology. Ke-CC: conceptualization and supervision. All authors designed and conducted the study, analyzed the data, and wrote the daft. All authors contributed to the interpretation of results, critically reviewed the draft, and approved the final manuscript.

## Conflict of Interest

The authors declare that the research was conducted in the absence of any commercial or financial relationships that could be construed as a potential conflict of interest.

## Publisher's Note

All claims expressed in this article are solely those of the authors and do not necessarily represent those of their affiliated organizations, or those of the publisher, the editors and the reviewers. Any product that may be evaluated in this article, or claim that may be made by its manufacturer, is not guaranteed or endorsed by the publisher.
